# Seeing with Different Eyes. The Module *Life & Science* of the Elite-Master Program Biomedical Neuroscience

**DOI:** 10.1007/s40670-024-01992-3

**Published:** 2024-02-06

**Authors:** Moritz Schumm, Daniel Teufel, Michael Brunnhuber, Marjo Wijnen-Meijer, Pascal O. Berberat

**Affiliations:** Technical University of Munich, TUM School of Medicine and Health – University Hospital rechts der Isar, TUM Medical Education Center (TUM MEC), Nigerstraße 3, Munich, Bavaria 81675 Germany

**Keywords:** Neuroscience, Philosophy, Anthropology, Interdisciplinarity, Professional identity formation, Critical thinking

## Abstract

From its beginnings in 2018, the international elite-master program *Biomedical Neuroscience* of *TUM School of Medicine and Health* at *Technical University of Munich* was guided by two convictions: First, excellent research depends not only on the mediation of scientific knowledge and skills, but also on a multifaceted understanding of science itself. Second, teaching must recognize and support not only student’s growing expertise but also their personal and professional development. To this end, the module *Life & Science* was designed. It gives students the opportunity to explore neuroscience from different angles to deepen the epistemological, sociological, and cultural understanding of their chosen profession.

## Introduction

In 1975, young sociologist Bruno Latour started his research at the endocrinological laboratory at *Salk Institute*, founded and lead by soon to be Nobel laureate Roger Guillemin. With the status of an intern, his mission was to have a keen look on a very curious tribe: scientists. The whole enterprise was guided by the idea that a look from a different angle would open up new insights into science and new ways of understanding its practice. Apart from the how-to knowledge of its practitioners and their habitualized daily routines, Latour’s position as an outsider allowed him a new approach to the ways of fact creation, data processing, and the negotiation as well as interpretation of results. And even though Jonas Salk himself grew skeptical towards the project, he still highlighted its core benefit. Delivering the introduction to the accompanying publication, he wrote: “[T]he book is not unworthy of the attention of scientists and the rest of society. […] One of their main points is that the social world cannot exist on one side and the scientific world on the other because the scientific realm is merely the end result of many other operations that are in the social realm.” [1].

Not unworthy indeed! The publication of *Laboratory life. The construction of scientific facts* (orig. publ. 1979) has become a highly influential source for the history of science and epistemology. And it is one of the most important founding documents of nowadays highly appreciated *actor-network theory* (ANT) [2, 3]. Besides its impact and one’s individual position towards its results, its core values lie, first, in showing that science itself is not a practice that is isolated from the rest of the world, but one that entertains—even if sometimes quite subtle—highly important relations to it. And this holds true not only for its rootedness in the “social realm”, but also for the broader areas of philosophy and culture as well as public discourse, history, and ideology. Second, Latour’s approach shows in its success the productivity of such additional knowledge and of a critical perspective on science. In its own respect, Latour’s research is an eloquent advocate for the advantages of critical thinking in general and—with its focusses on the laboratory and the discovery of the thyrotropin-releasing hormone TRF(H)—neuroscience in particular.

In a similar way, *Biomedical Neuroscience* at the *TUM School of Medicine and Health* of *Technical University of Munich* has followed from its very beginnings the conviction, that excellent research is not only relying on perfectly trained students and scientists. What is needed is a reflective and critical mindset that is able to situate scientific proceedings within the broader context of history, society, and epistemology [4–7]. Knowledge about the foundations and consequences of scientific research equips students with a heightened competence in dealing with challenging situations and assists them in taking on a well-founded responsibility in balancing research’s outlines, opportunities, and risks [8]. Understood also as a form of *professional identity formation* [9], it offers students a special outlook on their own position—their purpose as well as their aspirations—within neuroscience, enabling them to a more validated and reasoned decision-making in their research [10] as well as personal development.

To this end, the module *Life & Science* was created as an integral part of the master program *Biomedical Neuroscience* spanning two semesters with a total of 10 courses (each three hours). It is shaped out of a close collaboration between the elite-master program and the *LET ME* program of the *TUM Medical Education Center*. The latter—an abbreviation for *lettered medicine* or *lettered medical education*—is a special program for medical students. Its main focus lies on emphasizing and discussing key aspects of the medical profession and clinical everyday routine, that are not covered by regular medical teaching, but are nonetheless an integral part of medical student’s future line of work—for instance specific emotional challenges, finding one’s own role in medicine, or defining what it means to be a good doctor [11]. As these examples already suggest, the program is dealing predominantly with questions that defy a direct or generalizable solution, but ask for one’s personal attitude and individual determination. A focus that already comes close to the objectives set up for *Life & Science* and is further strengthened by a teaching framework and content that primarily derives from considerations directly out of philosophy and the humanities. Where the *LET ME* program is using works of art, literature, film, and philosophy to address its topics from a new and distinctly non-medical point of view to open up new perspectives, *Life & Science* is focused on topics straight out of epistemology and is entertaining a course structure that is concentrated on open-ended discussions without any pre-defined take home messages. Such a module, situated directly within the context of neuroscience, may seem therefore—at first glance—as a provocation. Because even if philosophy plays an integral part in a variety of different neuroscientific programs, the basic assumptions and subject matter of such a module still contrast highly with the otherwise taught set of scientific knowledge and skills. But the provocation is not only accepted but also welcomed for being a highly productive one that stimulates students to think about their studies, their roles, and their future profession in new, creative ways.

The article will elaborate on the special considerations and teaching objectives under which *Life & Science* is performed. Subsequently it describes the general structure and the incorporation of the module within the curriculum of *Biomedical Neuroscience*. A third section focuses on the content of the module and its specific didactic methods. These explanations will be accompanied by some examples of specific sessions to give further insight into the module’s discourses and their significance for *Biomedical Neuroscience* as well as for scientific research in general. A closing paragraph will give additional information on the evaluation of the module so far and its future perspectives.

## Special Considerations and Teaching Objectives

Three core considerations inform the module formally as well as content-wise. First, there is always an increase in complexity the closer something is observed. In its own way, what Karl Marx was coining for commodities also holds true for every phenomenon under close surveillance in that it “seems at first glance to be a self-evident, trivial thing. The analysis of it yields the insight that it is a very vexatious thing, full of metaphysical subtlety and theological perversities” [12]. Following this assumption, *Life & Science* exists to establish a critical look on neuroscience and its discourses. It wants to encourage its students to an attitude of critical thinking [13, 14] with the capacity to overcome an all to easily (or out of habitualization) accepted self-evidence of the world.

Therefore, and as the second consideration, there are no wrong, misplaced, or out of the order questions. On the contrary, the ostensibly “stupid questions” are key for our module. The laugh about such a question is in our context misplaced, because it acts as a simplistic affirmation of the naïve givenness of the world, whereas the “stupid question”—taken seriously—is really in search for an understanding of reality and its meaning. This is reflected in the two subtitles of our module. They designate each one of the module’s two semesters as two different parts of *Life & Science* in the form of two guiding questions: “What is life?” and “What is science?” These questions might themselves seem—at first glance—too simple for an elaborate discussion within an elite-master program. But what do we really and exactly mean when we talk about life? Is there a single, monologic definition without any opposition? Can one think of any approach that is not facing a variety of completely different ways of understanding? Putting emphasis on a functioning metabolism with an effective catalysis may give a scientific explanation but wouldn’t count much for cultural anthropology or the philosophic tradition of the “élan vital” [15].^1^ And a sole focus on life as a bodily phenomenon finds its epistemological difficulties in coping with theories concentrating on the mind. A comparable set of questions concerning science would take a different shape. But it would also lead directly from the simplicity of the phenomenon taken for granted to the mystery of a heterogeneous field of many different and contradictory solutions. Each solution bringing with it certain epistemological consequences that also have to be taken into account [16, 17].

All this leads to a further—and third—important aspect of the module’s teaching strategy: the topics of *Life & Science* are selected for their general insolvability. As much as life and science are basic concepts that are fundamental for scientific research in general, they cannot be pinned down in an epistemologically convenient way. No wonder they have a very long history in philosophy (and ideology) that is trying to clarify the phenomena, but offers instead a vast array of diverging perspectives and discourses. All of which are legitimate in their own rights, but highly contradictory in relation to each other. This is the point where the real provocation of the module lies: In an elite-master program, which’s main focus naturally relies on the scientific method to attempt the ideal of objectivity, *Life & Science* asks its students for their opinion, to form their own reasoned positions, and to take the “polyphony” of possible answers seriously [11]. The provocation reveals not only the demarcation and limits of scientific reasoning but also the cultural foundations of science that are themselves subject to the dynamics of history and discursive change [18].

## General Structure and Integration into the Curriculum of Biomedical Neuroscience

The module *Life & Science* stretches over the course of two semesters with a total sum of ten sessions: five per semester and each with a duration of three hours. Its course runs parallel to another module of the curriculum: *Scientific Practice*. As a second additional offer to the regular scientific teaching, *Scientific Practice* conveys a general set of skills that is applicable across all scientific subjects and disciplines. Parts of the module are methodological principles like deductive and inductive logic, scientific problem solving, and limitations of scientific inquiries as well as techniques of dissemination and ethical aspects. *Scientific Practice* and *Life & Science* as parallel modules represent together the section *Transferrable Skills* within the curriculum of *Biomedical Neuroscience*. And of course—as the term *Transferrable Skills* already suggests—both modules make good use of interdisciplinary synergies. While *Scientific Practice* is tailored for tackling more general principles of scientific work, *Life & Science* relies strongly on philosophical aspects. Together, they both share a special engagement in the teaching of skills and knowledge that are both not directly covered by neuroscientific lessons, but are nonetheless crucial for a successful research and career building. Finally, they also share a combined module at the end of the third semester called *Qualifying Colloquium*. After successfully passing *Scientific Practice* and *Life & Science*, tables are turned in this module. Here, students get the chance to present their master thesis to their colleagues and the course managers, paying special attention to the impact and the significance of both modules of *Transferrable Skills* on their own research.

The quality of this interdisciplinary approach in *Life & Science* is secured by its collaborative nature. The whole module is organized and held by a teaching assistant of the *LET ME* program with roots in the humanities. This course manager is accompanied for each session by an expert in the fields of philosophy, medicine, theory of science, or sociology. A great part of this accompaniment is carried out by professors from the *Munich School of Philosophy*, who are experts in the fields of natural philosophy, anthropology, metaphysics, as well as the philosophy of mind and of language. Additionally, a medical expert from the *TUM Medical Education Center* and—since 2020—a researcher from the *TUM School of Social Sciences and Technology* are joining specific sessions as guests.

The experts all take part in the sessions as participants with a special knowledge and expertise that they share during discussion with the students. This not only ensures a fruitful discussion on an advanced level, but also a well-balanced atmosphere: By not having a fixed presentation and instead taking part in the dynamics of the argument, the gap between experts and students is kept relatively small. Solely reacting on student’s inputs, the experts’ contributions are always kept very close to the interests and questions of the students.^2^ By these means, a very vivid and well-founded debate is ensured.

## Content and Specific Didactic Methods

Following the most crucial elements of the implementation of *Life & Science* in the elite-master program, concentration on the concrete composition of the course and its individual sessions is necessary. When the module *Life & Science* is structured by its two subtitles “What is Life?” and “What is Science?”, then its effects can be described as working in two directions.

First, in the very general direction of the superior question of the module: What is life science? The question is not central in respect to a definitive answer that is expected from the students at the end of the module. It is central as the question itself to provide students with an elaborate reflection of their studies, their future profession, and their personal role as researchers. If there is an answer, it should be understood as the expression of a personal consideration and individual choice. The result of the course is, in this sense, a thought through attitude that is reliable as well as instructive for one’s future career and research.

**Fig. 1:**
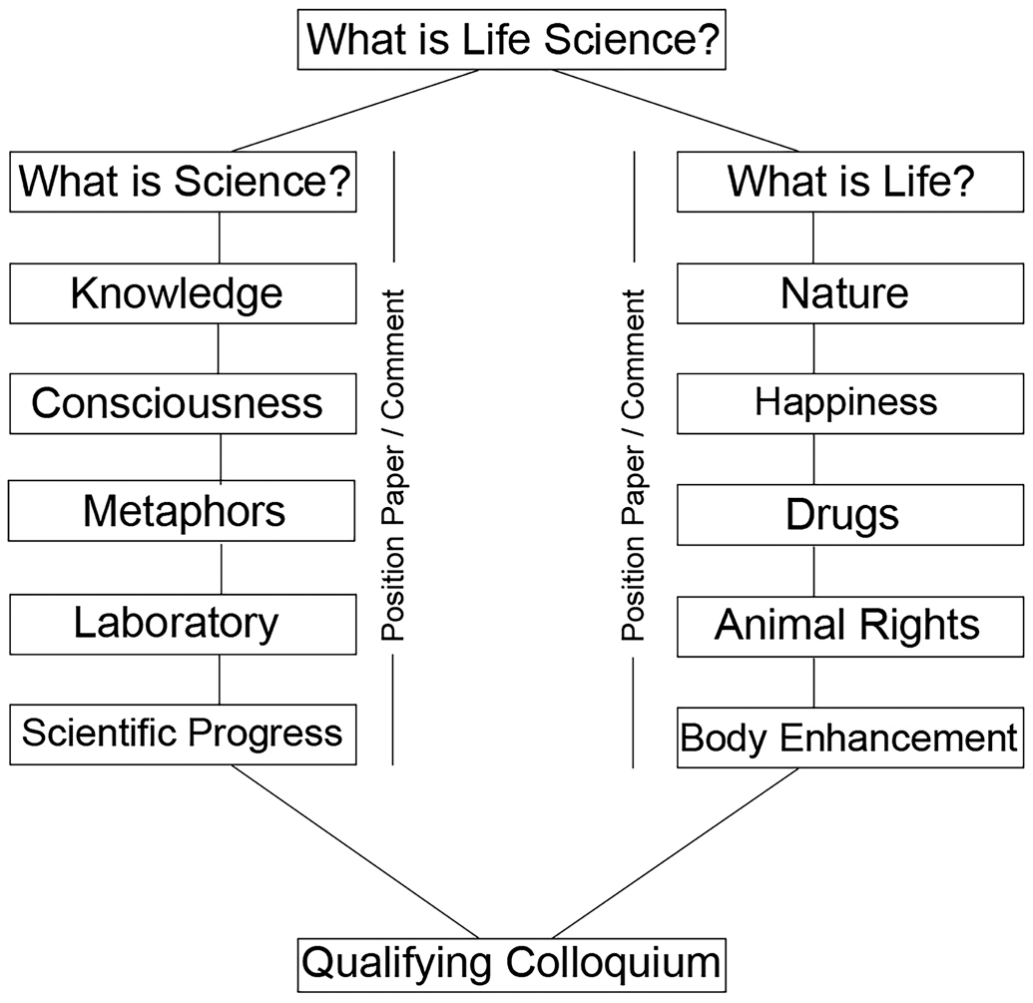
General structure of the module *Life & Science* within the elite-master program *Biomedical Neuroscience*

Second, the questions give structure to the five individual sessions that shape each of the two semesters of the module. Each session takes one important aspect of life or science as its main topic and elaborates on its specific attributes and meanings.^3^ Starting with the part “What is science?”, the main topics are “Knowledge”, “Consciousness”, “Metaphors”, “Laboratory”, and “Scientific progress”. The main themes of the following semester under the title “What is life?” are “Nature”, “Happiness”, “Drugs”, “Animal rights”, and “Body enhancement” (Fig. 1). Of course, a list of all the important aspects that can be considered when talking about life and science will never be complete. In this sense, the topics are chosen for covering the most important elements and because of their possible function as umbrella terms. Every term covers a variety of different meanings, so that its significance can shift in relation to the discussion’s dynamics during the session. All topics also share the characteristic, that they can be focused at first on the common problem of defining their respective terms or, more precisely, the phenomenon behind each one of them. The question “What is/are …?” could accordingly be added to each of the session’s main topics, in that the question is the ideal opener for the subsequent discussion. It not only opens up a heterogenic if not controversial and contradictory field of different perspectives but also highlights the most important elements for the students and their interests.

Talking, for example about “Nature” according to the explanations given by Thomas Henry Huxley’s “Nature: Aphorisms by Goethe” [19]—the very first article published in *Natur*e—offers a variety of different associations, metaphors, and definitions. And it is for a good part in the hands of the students in what direction they want to push the discussion. They can follow the more romantic pathway of the text that entertains a high reliance on the conviction that “everything speaks” [20] as a fundamental trade of nature. They can take on a critical, investigative attitude by following the text’s rejection of an anthropomorphizing of nature. They can criticize the text itself for its blunt poetics. Especially when told the fact, that the attribution of the extensive quote to Goethe is—most probably—incorrect.^4^ Or, on the contrary, they can follow the poetic traits of the descriptions and discuss their very own truths, when metaphors like “mother” or “circle” are legion and have their own specific way of characterizing the experience of nature.

As a starting point for each session, students have to read one or two articles in preparation for each session. The theme of the respective article lays out central characteristics of the topic—its individual problematization as well as its possible meaning and importance for students. The texts can be instructive and offer guidelines for the discussion. But they can also take on the form of controversial statements that are open to critique from the students. Reading, for example, excerpts from George Lakoff’s and Mark Johnson’s *Metaphor’s we live by* [21] is in itself first and foremost a highly productive introduction to the topic of linguistics, metaphors, and their impact on everyday as well as scientific thinking. Reading—in contrast—a chapter from Yuval Noah Harari’s *Homo deus. A brief history of humankind* [22] is in its broad generalizations and often rather crude theorization still an excellent basis for discussion. In its own and more confrontative manner, it supports a lot of different positions towards and against Harari’s point of view that make for a very lively and intriguing discussion.

For both parts of the module, students also have to write a short position paper (approximately 5000 characters) on one of the five topics. The papers must be submitted at least one week ahead of the respective session. They should contain a short summary of the text accompanying the respective topic and a short elaboration of one’s own point of view towards the subject. Finally, it should state at least one question that should be discussed during the session. These questions help clarifying what aspects are most important for the students. Directly asked, they also give an outline for the structure of the session. This once again helps ensuring a discussion that is close to student’s interests and their perspective as scientists.

Further, each student has to submit a more extensive comment (approximately 15,000 characters) on one of the sessions in retrospect (no later than two weeks after the discussion) that is meant to be a more elaborated presentation of student’s thoughts about the topic and the specific aspects that are relevant to them. Position paper and comment are both graded individually by the module responsible. Together with a regular attendance their cross-section represents the final grade of the module.

Besides the questions from the students, the sessions are structured by impulses given by the course manager. These can consist of a quotation, a piece of art, pop culture, or film and video. They are chosen for highlighting specific aspects of the respective topic and to engage with them from another angle. Via the analysis of a short text, picture, or film clip, special constellations, details, or ideas can be discovered and transferred to the general topic and its discussion. For example, in the case of the session concentrating on metaphors and their significance for everyday life as well as scientific practice, the analysis of René Magritte’s famous Painting *La trahison des images* (1929) with its depiction of a pipe and the written statement “Ceci n’est pas une pipe” (“this is not a pipe”) has proven to be very enlightening. Its bold play with images and words contradicting each other is questioning the very nature of language and pictorial representations. Its dilemma opens up new perspectives on science as a discipline that itself is relying heavily on the possibilities of writing and image-generating devices—both of which with their own epistemological and ontological ambivalences.

One last, but especially important element in structuring the sessions of *Life & Science* is the use of writing exercises. At the beginning and at the end of each session, students are given a specific question related to the topic as a prompt. They are asked to take five minutes to reflect on it and write down an answer. The importance lies in a once again new angle on the topic, but this time in the form of a creative occupation and a fixed result that outlasts the otherwise transitory and ephemeral discussions. As opening and closing statements, the developed texts are the concentrated results of the session that are read aloud by each student for the whole class. In this way, they open up a panorama of the different perspectives and possible discourses that are taking part in the discussion. The session on Happiness, for example, starts with the questions: “When was the last time you felt really happy? Why?” This not only relates the current topic to one’s own lived experience. It also and already presents a variety for different instances and forms of articulation of happiness which can lead directly into the discussion.

## Evaluation and Perspectives

Like Bruno Latour’s pioneering work, *Life & Science* puts—as a teaching method—sociology, humanities, and philosophy in the laboratory and classroom of (future) neuroscientists. It works—just like Latour’s approach—not least, because of its quality as a *Verfremdungseffekt* (alienation effect). The term was coined by German playwriter Bertolt Brecht for his Epic Theater [23]. Its main idea is to break the self-evident givenness of a situation by introducing a foreign element to it that questions the whole scene. The result is, as the German philosopher Walter Benjamin put it, a “‘[t]ableau’, as they used to say around 1900” [24] that allows a fresh and new outlook on otherwise well-known circumstances.

By the end of the summer term 2023, the module has completed its fifth cycle. Evaluation via questionnaires has shown in average a high approval of the course’s content and teaching methods. Existing evaluations^5^ show that in particular the motivating design and the stimulating atmosphere were very well received (by an average of approximately 73%). Further, approximately 72% of the students agreed that the module made them reflect on the respective topics. Concerning the general connection of the module’s topics to scientific practice approximately 63% agreed that the course was an important addition to their study. Considering the unusual content and structure of this mandatory course, the still significant minority of 37% that sees a gradually lesser applicability was to be expected. At the same time, the comment section of the questionnaires highlighted the general approval of the module and its topics. *Life & Science* was described as an “important addition” that gives “[t]he chance to discuss more philosophical topics” and adds “great value to the otherwise very scientific master-program.” Besides that, the overall workload in *Life & Science* received a critical reception. Still 66% agreed to its feasibility. But the comments in the evaluation pointed out, that the schedule of *Life & Science*—taking place in the evening with a high amount of other courses before—could be reconsidered. It was also stated that the comments put considerable weight on the already fully occupied schedule.

Understood as a principal encouragement of the work done so far, *Life & Science* is looking forward to its future sessions. With a growing number of students an alignment of the written tasks is part of current considerations. Further, a closer collaboration of *Life & Science* with *Scientific Practice* is in planning. A stronger combination of the *Transferrable Skills* is estimated to offer some improvement for the general time management. At the same time, its main benefit will be found in a much stronger exploit of the synergetic potentials for the students.

## Data Availability

Data represented in this paper is available upon request to the corresponding author.

## Footnotes

1 Closely related to Henri Bergson’s idea of durée, the term is aiming at an evolutionary understanding which’s development is guided at every instance by a certain form of consciousness as well as intentionality.

2 Consisting of approximately 15 students each year, the relatively small number of participants is also contributing to a productive discussion.

3 Some changes have been made over the years to adapt the topics further to students’ interests. This list details the curriculum of *Life & Science* as it is taught at the moment of submission.

4 The text Huxley’s referring to as being written by Johann Wolfgang Goethe is nowadays attributed to Georg Christoph Tobler.

5 The results stated here are retrieved from the questionnaires of the first, fourth, and fifth group of students who concluded the *Life & Science* curriculum. During the COVID pandemic, evaluation was suspended.
